# Recurrent cerebral attack caused by thrombosis in the pulmonary vein stump in a patient with left upper lobectomy on anticoagulant therapy: case report and literature review

**DOI:** 10.1186/s40792-017-0376-1

**Published:** 2017-09-11

**Authors:** Takahito Nakano, Mayumi Inaba, Hiroyuki Kaneda

**Affiliations:** 1 Department of Thoracic Surgery, Kansai Medical University Medical Center, 10-15 Fumizonocho, Moriguchishi, Osaka 570-8507 Japan; 2 Department of Diagnostic Pathology, Kansai Medical University Medical Center, 10-15 Fumizonocho, Moriguchishi, Osaka 570-8507 Japan

**Keywords:** Lung cancer, Pleomorphic carcinoma, Left upper lobectomy, Pulmonary vein thrombosis, Cerebral infarction, Anticoagulation therapy

## Abstract

**Background:**

Thrombus formation in the pulmonary vein stump after pulmonary resection has recently been identified as a cause of systemic thrombosis including brain infarction. However, there is limited research focusing on the clinical course of pulmonary vein stump thrombus, and optimal treatment and prevention strategies of this important complication have not been established.

**Case presentation:**

A 77-year-old woman was diagnosed with lung cancer of the left upper lobe, cT4N2M0, cStage IIIB. As the tumor was considered to be completely resectable, the patient underwent a left upper lobectomy with angioplasty of the left pulmonary artery. The final pathological stage was pT4N2M0, pStage IIIB. The patient developed paralysis of the right upper limb and dysarthria on the 8th postoperative day. Diffusion-weighted magnetic resonance imaging (MRI) of the brain showed multiple high-intensity signals in the area of the left middle cerebral artery, which were not detected on preoperative MRI. She was diagnosed with a cerebral infarction and started on acute-phase treatment including anticoagulation with continuous intravenous heparin infusion. The neurological symptoms improved the following day. Contrast-enhanced chest CT scan revealed thrombus in the left superior pulmonary vein stump measuring 10 mm in diameter. She had no comorbidity related to the cerebral attack. After the treatment was initiated, her symptoms became stable. However, symptoms of altered consciousness, dysarthria, and hemiparesis re-occurred on the 19th postoperative day and improved within an hour. The thrombus in the left superior pulmonary vein stump disappeared on follow-up contrast-enhanced chest CT performed the same day.

**Conclusions:**

This is the first report of recurrent brain attack caused by thrombosis in the pulmonary vein stump in a patient receiving anticoagulant therapy. The present case suggests the possibility of thrombus mobilization causing recurrent systemic thrombosis, and this important complication needs to be considered in future clinical practice.

## Background

Thrombus formation in the pulmonary vein stump after pulmonary resection has recently been identified as a cause of systemic thrombosis including brain infarction [1]. However, there is limited research focusing on the clinical course of pulmonary vein stump thrombus, and optimal treatment and prevention strategies of this important complication have not been established. Herein, we report a case of recurrent brain attack caused by thrombosis of the pulmonary vein stump in an anticoagulated patient after left upper lobectomy and include a review of previous literature.

## Case presentation

**Fig. 1:**
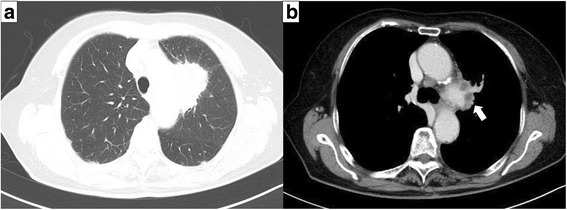
Preoperative chest computed tomography (CT) scan. **a** Chest CT scan showing a 5 cm tumor in the *left upper lobe*. **b** Tumor was suspected to invade the pulmonary artery (*arrow*)

**Fig. 2:**
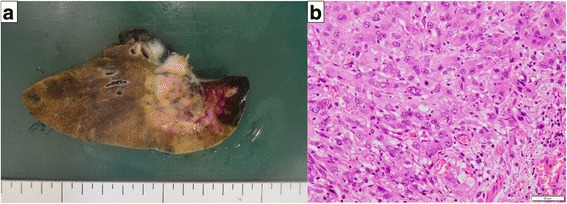
Pathological findings. **a** Resected specimen showing a 6.6 cm × 4.3 cm-sized tumor in the *left upper lobe*. **b** Histological type was pleomorphic carcinoma containing a variable adenocarcinoma component

A 77-year-old woman presented with hoarseness. A chest computed tomography (CT) scan showed a tumor with a diameter of 5 cm in the left upper lobe suspected to involve the mediastinal lymph nodes and invade the pulmonary artery (Fig. 1). An 18-fluoro-2-deoxyglucose-positron emission tomography scan demonstrated that the maximum standardized uptake value at the center of the tumor was 22.0. Bronchoscopic biopsy results confirmed the diagnosis of lung adenocarcinoma (cT4N2M0, cStage IIIB). As the patient showed symptoms of bloody sputum and the tumor was considered to be completely resectable, she underwent a left upper lobectomy with angioplasty of the left pulmonary artery. The intraoperative volume of blood lost was 187 ml and the volume of infusion was 2650 ml. Oral intake was restarted the next morning. Pathological evaluation revealed that the tumor size was 6.6 cm in maximum diameter, and it invaded the pulmonary artery. The histological type was pleomorphic carcinoma containing a variable adenocarcinoma component (Fig. 2). The final pathological stage was pT4N2M0, pStage IIIB.

On the second postoperative day, her electrocardiogram showed a high frequency of ventricular premature complexes and the echocardiograph indicated Takotsubo cardiomyopathy. She was treated with human atrial natriuretic peptide, and anticoagulant therapy with continuous intravenous heparin infusion was administered to prevent intracardiac thrombus formation. Activated partial thromboplastin time (APTT) was under 50 s. During the treatment, echocardiography did not detect intracardiac thrombus formation, and anticoagulation therapy was withdrawn on the 7th postoperative day.

**Fig. 3:**
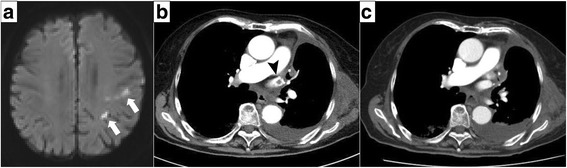
**a** Diffusion-weighted magnetic resonance image (MRI) of the brain showing multiple high-intensity signals in the area of the left middle cerebral artery (*arrow*). **b** Contrast-enhanced chest CT after the first episode of cerebral infarction showing a thrombus in the *left* superior pulmonary vein stump measuring 10 mm in diameter (*arrow head*). **c** Thrombus in the left superior pulmonary vein stump disappeared on follow-up contrast-enhanced chest CT scan after the second episode of cerebral infarction

The patient developed paralysis of the right upper limb and dysarthria on the 8th postoperative day. Diffusion-weighted magnetic resonance imaging (MRI) of the brain showed multiple high-intensity signals in the area of the left middle cerebral artery, which were not detected on preoperative MRI (Fig. 3a). She was diagnosed with a cerebral infarction and started on acute-phase treatment including anticoagulation with continuous intravenous heparin infusion. APTT was maintained over 60 s. The neurological symptoms improved the following day. Contrast-enhanced chest CT scan revealed thrombus in the left superior pulmonary vein stump measuring 10 mm in diameter (Fig. 3b). She had no comorbidity related to the cerebral attack. To exclude other causes of thrombosis, we performed magnetic resonance angiography, which revealed no significant findings in the carotid artery.

After the treatment was initiated, her symptoms became stable. However, symptoms of altered consciousness, dysarthria, and hemiparesis re-occurred on the 19th postoperative day and improved within an hour. The thrombus in the left superior pulmonary vein stump disappeared on follow-up contrast-enhanced chest CT performed the same day (Fig. 3c). After the second cerebral event, long-term treatment with apixaban, a direct oral anticoagulant, was initiated. At 3 months after surgery, the patient had no more recurrent embolic events.

## Discussion

Thrombus formation in the stump of the pulmonary vein after left upper lobectomy is a risk factor for embolism to vital organs and can result in brain infarction, renal infarction, myocardial infarction, and internal carotid artery occlusion [1–5]. Ohtaka and colleagues [6] reported the frequency and risk factors for thrombus formation after lobectomy for lung cancer and found that a thrombus in the pulmonary vein stump was detected in 3.6% of all patients that underwent lobectomy and in 13.5% of those that underwent left upper lobectomy. Univariate analyses revealed that left upper lobectomy and operative time were notable risk factors for thrombus formation.

We described the case of a patient with two episodes of cerebral infarction after left upper lobectomy despite the fact that she was on anticoagulation therapy. Thrombus in the left superior pulmonary vein stump was detected at the first episode of cerebral infarction, but it disappeared at the second cerebral event. A possible explanation for this finding may be that, after it decreased in size, the thrombus was released from the pulmonary vein stump into systemic circulation due to the anticoagulation therapy and subsequently caused a recurrent cerebral infarction.

We performed a literature review with the PubMed and Japan Medical Abstracts Society databases with the following search terms: thrombus, pulmonary vein stump, left upper lobectomy, and systemic thrombosis. To date, 32 cases have been reported (Table 1). Of the 32 cases, cancer stages were described in 19 cases, including 10 cases of stage I, 5 of stage II, and 4 of stage III. Histologic types were described in 14 cases, including 10 cases of adenocarcinoma, 2 of squamous cell carcinoma, and 2 of other histologic types. Of the 32 cases reviewed, 24 of the patients received anticoagulation therapy and the thrombus disappeared in most cases (Table 2).

**Table 1:** Reported cases of thrombus formation in the pulmonary vein stump after pulmonary resection

Authors (year)	No. of patients	Procedure of pulmonary resection (*n*)	Site of thrombosis (*n*)
Ohtaka (2013) [6]	7	LUL (7)	Occult (6), brain (1)
Ohtaka (2014) [8]	2	LUL (2)	Occult (2)
Ohtaka (2014) [9]	5	LUL (5)	Occult (5)
Ichimura (2014) [10]	4	LUL (4)	Occult (3), kidney (1)
Yamamoto (2016) [7]	3	LUL (3)	Brain (3)
Other case reports [2–5, 11–17]	10	LUL (8), LUS (1), RLL (1)	Brain (5), occult (2), kidney (2), leg (1)
Present case	1	LUL (1)	Brain (1)
Total	32	LUL (30), LUS (1), RLL (1)	Occult (18), brain (10), kidney (3), leg (1)

Patients in different reports by Ohtaka [6, 8, 9] were different series

*LUL* left upper lobectomy, *RLL* right lower lobectomy, *LUS* left upper division segmentectomy

**Table 2:** Treatment for thrombus in the pulmonary vein stump and outcomes of reported cases

Treatment	No. of patients	Thrombus after treatment (*n*)	Systemic thrombosis after treatment
Anticoagulation therapy	24	Disappeared (17), decreased in size (1), ND (6)	None (24)
Surgically removed	2	Disappeared (2)	None (2)
Observation	1	Disappeared (1)	None (1)
Died before treatment	1		
ND	3		

*ND* not described

There were two patients that developed a first episode of thrombosis despite being on anticoagulant therapy [2, 7]. One patient [2] was taking anticoagulation therapy at baseline because of atrial fibrillation and developed thrombosis on the 50th postoperative day. The patient was receiving adjuvant chemotherapy, which may be a risk factor for thrombus formation as indicated in a previous report [6]. Another patient [7] was taking antiplatelet therapy at baseline, and this medication was preoperatively bridged to continuous intravenous heparin infusion and stopped 6 h before surgery. The patient underwent left upper lobectomy and continuous intravenous heparin infusion was restarted 2 h after surgery, but cerebral infarction occurred the next day after surgery.

Anticoagulation therapy seems to be effective to decrease the thrombus and prevent thrombosis formation. The present case suggests the possibility of thrombus mobilization causing recurrent systemic thrombosis. We should consider the risk of thrombosis under anticoagulant setting. The benefits and risks of treatment for thrombus in the pulmonary vein stump need to be clarified in a prospective study.

## Conclusions

This is the first report of recurrent brain attack caused by thrombosis in the pulmonary vein stump in a patient receiving anticoagulant therapy. In our review of the literature, anticoagulation therapy seems to be effective to remove the thrombus and prevent thrombosis. The present case suggests the possibility of thrombus mobilization causing recurrent systemic thrombosis, and this important complication needs to be considered in future clinical practice.

## Abbreviations

APTT: Activated partial thromboplastin time
CT: Computed tomography
MRI: Magnetic resonance image
